# Expression profiles and functional prediction of long non-coding RNAs LINC01133, ZEB1-AS1 and ABHD11-AS1 in the luminal subtype of breast cancer

**DOI:** 10.1186/s12967-021-03026-7

**Published:** 2021-08-26

**Authors:** Sepideh Mehrpour Layeghi, Maedeh Arabpour, Abbas Shakoori, Mohammad Mehdi Naghizadeh, Yaser Mansoori, Javad Tavakkoly Bazzaz, Rezvan Esmaeili

**Affiliations:** 1 Department of Medical Genetics, School of Medicine, Tehran University of Medical Sciences, Tehran, Iran; 2 Medical Genetic Ward, Imam Khomeini Hospital Complex, Tehran University of Medical Sciences, Tehran, Iran; 3 Breast Disease Research Center (BDRC), Tehran University of Medical Sciences, Tehran, Iran; 4 Noncommunicable Diseases Research Center, Fasa University of Medical Sciences, Fasa, Iran; 5 Department of Medical Genetics, Fasa University of Medical Sciences, Fasa, Iran; 6 Genetics Department, Breast Cancer Research Center, Motamed Cancer Institute, ACECR, Tehran, Iran

**Keywords:** Breast cancer, LINC01133, ZEB1-AS1, ABHD11-AS1, Luminal A, Luminal B, Bioinformatics analyses

## Abstract

**Background:**

Luminal breast cancer (BC) is the most frequent subtype accounting for more than 70% of BC. LncRNAs, a class of non-coding RNAs with more than 200 nucleotides, are involved in a variety of cellular processes and biological functions. Abberant expression is related to the development of various cancers, such as breast cancer. LINC01133, ZEB1-AS1, and ABHD11-AS1 were reported to be dysregulated in different cancers. However, their expression level in luminal BC remains poorly known. The aim of the present study was to evaluate the potential roles of these lncRNAs in BC, especially in luminal subtypes.

**Methods:**

A comprehensive analysis was performed using the Lnc2Cancer database to identify novel cancer-associated lncRNA candidates. After conducting a literature review, three novel lncRNAs named LINC01133, ZEB1-AS1, and ABHD11-AS1 were chosen as target genes of the present study. Quantitative real‐time polymerase chain reaction (qRT-PCR) was used to evaluate the expression level of the mentioned lncRNAs in both luminal BC tissues and cell lines. Then, the correlation of the three mentioned lncRNAs expression with clinicopathological characteristics of the patients was studied. Moreover, several datasets were used to discover the potential roles and functions of LINC01133, ZEB1-AS1 and ABHD11-AS1 in luminal subtype of BC.

**Results:**

According to the qRT-PCR assay, the expression levels of LINC01133 and ZEB1-AS1 were decreased in luminal BC tissues and cell lines. On the other hand, ABHD11-AS1 was upregulated in the above-mentioned samples. The expression levels of LINC01133, ZEB1-AS1, and ABHD11-AS1 were not associated with any of the clinical features. Also, the results obtained from the bioinformatics analyses were consistent with qRT-PCR data. Functional annotation of the co-expressed genes with the target lncRNAs, protein–protein interactions and significantly enriched Kyoto Encyclopedia of Genes and Genomes (KEGG) pathways across luminal BC were also obtained using bioinformatics analysis.

**Conclusions:**

Taken together, our findings disclosed the dysregulation of LINC01133, ZEB1-AS1, and ABHD11-AS1 in luminal BC. It was revealed that LINC01133 and ZEB1-AS1 expression was significantly downregulated in luminal BC tissues and cell lines, while ABHD11-AS1 was upregulated considerably in the mentioned tissues and cell lines. Also, bioinformatics and systems biology analyses have helped to identify the possible role of these lncRNAs in luminal BC. However, further analysis is needed to confirm the current findings.

**Supplementary Information:**

The online version contains supplementary material available at 10.1186/s12967-021-03026-7.

## Introduction

Breast cancer (BC) is the most common cancer among women worldwide, accounting for 30% of all female cancers alone [1, 2]. It is also the second leading cause of cancer death with 11.6% of the total cancer deaths [3]. The luminal subtype of BC, expressing both estrogen and progesterone receptors (ER^+^/PR^+^), accounts for a large percentage of this cancer (more than 70%) [4, 5]. Luminal type of BC is classified into two groups, luminal A and luminal B, according to human epidermal growth factor receptor 2 (HER2) status and levels of ki-67 [5]. The leading choice for luminal BC treatment is endocrine therapy. However, resistance to endocrine therapy is a major challenge for clinicians [6]. Also, similar treatment strategies can provide a variety of responses in luminal subtype patients [7]. In general, despite the better prognosis, due to the heterogeneity and resistance to hormone therapy of luminal subtype, the treatment effectiveness is still limited [7]. Therefore, identifying novel biomarkers and molecular mechanisms of BC is required to perform early diagnosis and personalized therapeutic strategies. In addition, the limited number of studies on the dysregulation of long non-coding RNAs (lncRNAs) in luminal BC, as well as the high frequency of this subtype, increase the importance of further research on this subgroup.

The GENCODE project results have indicated that only about 2% of the human genome encodes proteins, whereas the vast majority of it is transcribed into non-coding RNAs [8]. LncRNAs, a class of non-coding RNAs with more than 200 nucleotides, have been shown to play essential roles in many different cellular processes, such as genetic imprinting, transcriptional responses, development, etc. [9, 10]. Therefore, the aberrant expression of lncRNAs can lead to the pathogenesis of various diseases including cancers [11]. They have been identified as crucial molecules involved in cancer proliferation, invasion, and resistance to therapy [12, 13]. Some lncRNAs are expressed highly specific in different cancer types, and some of them are associated with the survival of patients. These two features make them ideal prognostic and diagnostic tools [14]. Due to the development of lncRNA-based therapeutic approaches, determining the role of different lncRNAs in the tumorigenic process is of great importance. Hence, in the present study, we aimed to figure out the expression level and the underlying molecular mechanisms of LINC01133, ZEB1-AS1, and ABHD11-AS1 in luminal BC.

LINC01133, a novel lncRNA, is located on chromosome 1q23.2. It was reported that LINC01133 exerted a tumor suppressive role in gastric cancer [15], ovarian cancer [16], and esophageal squamous cell carcinoma [17] progression. It was also found that LINC01133 played oncogenic role in the development of some other cancers [18–20]. These dual roles of LINC01133 could attribute to the tissue-specific expression of this lncRNA. ZEB1-AS1 is associated with the progression and development of several cancers. Its aberrant expression was detected in many tumors [21], such as gastric cancer [22], hepatocellular carcinoma [23], and glioma [24]. ABHD11-AS1, which is located at 7q11.23, is a newly discovered lncRNA. Previous studies have reported the upregulation of this lncRNA in different tumors, including papillary thyroid carcinoma [25] and pancreatic cancer [26]. To the best of our knowledge, the present research is the first study that determines the dysregulation of ABHD11-AS1 in BC so far. Despite all the studies that have been done on these lncRNAs, their biological processes and potential mechanisms, specifically in luminal subtype of BC, are still unclear.

In the present study, we examined the expression level of three lncRNAs in luminal BC by qRT-PCR assay. Furthermore, we performed a correlation analysis between the expression of these lncRNAs and clinicopathological parameters of patients. Finally, to investigate the potential role and mechanism of the lncRNAs in luminal BC, different bioinformatics and systems biology analyses were accomplished using various databases.

## Materials and methods

### Identification of novel lncRNAs and performing literature mining

Lnc2Cancer database was used to discover novel lncRNAs that are associated with different types of cancers. Lnc2Cancer is a comprehensive database which provides lncRNA-cancer correlations between 165 different cancer types and 1614 lncRNAs [27, 28]. Then, to find lncRNAs that have been less or not studied in breast cancer, a comprehensive PubMed data mining was performed on the candidate lncRNAs. The following keywords were searched: “long non-coding RNA” or “lncRNA”, “breast cancer” or “breast carcinoma”, and “the candidate lncRNAs”. LncRNAs whose expression and role in luminal BC were not studied were selected. Also, among the selected lncRNAs, those that played an important role in several cancer-associated signaling pathways were included in the study.

### In-vitro analysis

#### Breast cancer tissue samples

To perform the present case–control study, luminal A and B BC tissue samples and paired adjacent non-cancerous tissues were obtained from 79 patients at Breast Cancer Research Biobank (BCRC-BB) [29]. BCRC-BB follows every ethical guideline about storing and utilizing human biological samples for biobanks. Tissue specimens were snap-frozen in liquid nitrogen and stored at − 80 °C until performing tests. The inclusion criterion was that patients should not receive any local or systemic treatment before surgery. Clinicopathological parameters including age at diagnosis, tumor size, tumor subtype, grade, stage, pathology of tumors, and ER/PR/HER2 status were retrieved from the hospital records. Written consent was signed by all of the patients, and all the samples were approved by relevant pathologists. Also, the Ethics committee of Tehran University of Medical Sciences (TUMS) approved the present research (Code: IR.TUMS.MEDICINE.REC.1398.792).

#### Breast cancer cell lines and culture

Luminal cell lines, MCF7 and T47D, and a non-invasive breast epithelial cell line, MCF10A, were used for the current project. MCF7 and T47D were grown in DMEM (Sigma-Aldrich, St. Louis, MO, USA), 10% fetal bovine serum (Gibco, Carlsbad, CA, USA), 100 U/ml penicillin, and 100 μg/ml streptomycin (Sigma-Aldrich, St. Louis, MO, USA). MCF10A were maintained in DMEM supplemented with 5% fetal bovine serum, epidermal growth factor (EGF) (20 ng/ml), insulin (10 µg/ml), hydrocortisone (0.5 mg/ml), cholera toxin (100 ng/ml), penicillin (100 U/ml), and streptomycin (100 μg/ml). An incubator at 37 °C with 5% CO_2_ and 95% humidity was required for maintaining cells.

#### Isolation of total RNA and qRT-PCR

Total RNA isolation from breast tissues and cultured cell lines was performed by RiboEx™ (GeneAll), applying the manufacturer’s instructions. Then, complementary DNA (cDNA) synthesis was carried out by performing reverse transcription process of 1 µg of isolated RNA samples using 5× All-In-One RT MasterMix kit (Applied Biological Materials). Next, q-RT PCR was carried out by using AMPLIQON Real Q Plus 2× Master Mix Green Low ROX on LightCycler96 Roche system. Moreover, using serially diluted samples, the efficiencies for each pair of primers of the genes of interest were calculated. The expression levels of the genes of interest were normalized to the expression level of Beta-2-Microglobulin (β2M) as an internal control. The primer sequences are listed in Table 1.

**Table 1:** PCR primer sequences

Genes	Forward (5′–3′)	Reverse (5′–3′)
β2M	AGATGAGTATGCCTGCCGTG	GCGGCATCTTCAAACCTCCA
LINC01133	GGGGAGAGTAGGTGAAAAGATGA	GCTGGACTTTGGAGAACTTTGC
ZEB1-AS1(T5,6)^a^	TGCATGAAGGTGGTATGGACT	GGGGTAGGAATAGGGATAACTGT
ZEB1-AS1(T1-4)^a^	CCTGTACCTCCCTGCTAAGC	GCCCAAACTAACTAAACCAGAAAC
ABHD11-AS1	CTCCAGGAACGGGATGAAG	CAGCCTCAGTTTCTCCTCCA

*β2M* Beta-2-Microglobulin, *PCR* polymerase chain reaction

^a^Two pairs of primers, namely ZEB1-AS1(T5,6) and ZEB1-AS1(T1-4), were designed to evaluate the expression level of different transcript variants of lncRNA ZEB1-AS1

### Bioinformatics analysis

Several web servers and databases were used to select the genes of the current study and their role in luminal BC (Table 2).

**Table 2:** Bioinformatics tools used to analyze the functions of LINC01133, ZEB1-AS1, and ABHD11-AS1 in BC

Database	URL	References
Lnc2Cancer database	http://www.bio-bigdata.net/lnc2cancer	[27, 28]
UCSC Xena	http://xena.ucsc.edu/	[30]
GDC	https://portal.gdc.cancer.gov	[31]
TANRIC	https://www.tanric.org	[32]
GENEVESTIGATOR	https://genevestigator.com	[33]
ICGC	https://dcc.icgc.org	[34]
DAVID	https://david.ncifcrf.gov	[35, 36]
REVIGO	http://revigo.irb.hr	[37]
Cytoscape	http://www.cytoscape.org	[38]
CytoHubba	http://apps.cytoscape.org/apps/cytohubba	[39]
STRING	https://string-db.org	[40]
Enrichr	http://amp.pharm.mssm.edu/Enrichr	[41]

#### Gene correlation analysis

While lncRNAs have constituted a rapidly expanding field of research, only a small number of them has been identified functionally thus far. Analyzing the mechanisms of co-expressed genes with lncRNAs can be very useful in predicting the potential function of novel RNAs. For this purpose, the UCSC Xena Browser [30] was used to select a list of luminal samples. The following parameters were included: “Luminal A”, “Luminal B”, and “The TCGA Sample ID”. Then, the TCGA ID of the specimens was used to search the FPKM file of the selected samples in the Genomic Data Commons (GDC) database [31]. Finally, a correlation analysis was performed to obtain the co-expressed genes with LINC01133, ZEB1-AS1, and ABHD11-AS1 across the selected samples. Pearson correlation coefficient for the co-expressed genes has been computed by a standard method (*R* ≥ 0.4).

#### Expression analysis among molecular subtypes of BRCA

The expression pattern of the lncRNAs LINC01133, ZEB1-AS1 and ABHD11-AS1 was further evaluated using TANRIC database [32] across TCGA Breast invasive carcinoma (BRCA) dataset. The analysis was performed among different BC subtypes of the PAM50 classification (Normal-like, Luminal A, Luminal B, Basal and HER2^+^). TANRIC database has characterized the expression pattern of several lncRNAs using TCGA and different independent datasets (> 8000 samples overall). In addition, the expression level of the mentioned lncRNAs was evaluated among luminal and also non-luminal cell lines using GENEVESTIGATOR database [33]. GENEVESTIGATOR integrates thousands of RNA-Seq and microarrays data in order to evaluate gene expression among several cancers.

#### Oncogenomic analysis

The International Cancer Genome Consortium (ICGC) portal [34] was used to identify genomic alterations of the selected lncRNAs across luminal BC. The mutational data was obtained from BRCA-EU project including ER^+^ HER2^−^ tumor subtypes (luminal A and B groups). Also, figures of the lncRNAs were illustrated by trackViewer R Package [42] according to the location of the mutations.

#### Functional enrichment analysis

To investigate the associated pathways and functions of LIN01133, ZEB1-AS1 and ABHD11-AS1, functional enrichment analysis of the genes co-expressed with these three lncRNAs was performed using various databases. First, the Database for Annotation, Visualization and Integrated Discovery (DAVID) [35, 36] was used for Gene Ontology (GO) term enrichment analysis. To this end, the list of co-expressed genes with LINC01133, ZEB1-AS1 and ABHD11-AS1 was used which was obtained in the previous step. Then, to remove redundant GO terms based on similar measures, the list of GO terms obtained from DAVID was submitted to the REVIGO [37]. At this level, Cytoscape software [38] was utilized for performing a detailed visualization of the networks specified by REViGO. The enrichr database [41] was also used to evaluate pathways in which three lncRNAs may be involved. Calculating and visualizing protein–protein interactions (PPI) of the co-expressed genes with LINC01133, ZEB1-AS1 and ABHD11-AS1 were done using the STRING database [40]. Also, the hub genes of the lncRNAs co-expressed genes were explored by the degree method available in CytoHubba [39], a Cytoscape application.

### Statistical analysis

IBM SPSS Statistics (Statistical Package for the Social Sciences, Version 24.0) was used for analyzing the data obtained from the qRT-PCR assay. The tumoral and adjacent non-tumoral tissues were extracted from paired sources. The differences between ∆Ct values of LINC01133, ZEB1-AS1, and ABHD11-AS1 expression levels in tumor and adjacent non-tumor tissue specimens were analyzed using paired sample t-test. For clinicopathological correlation analysis, the median expression of the lncRNAs was used for dividing 79 patients into two categories: relatively high and relatively low expression of the lncRNAs. Also, correlation analysis of LINC01133, ZEB1-AS1, ABHD11-AS1 and clinicopathological features was carried out by the chi-square and independent t-test. Moreover, Kaplan–Meier method and log-rank test were used to calculate the overall survival rates and compare differences between survival curves, respectively. The data is represented as mean ± standard deviation (SD). P-values of less than 0.05 were considered significant. The diagnostic value of each gene was calculated via the receiver operating characteristic (ROC) curve analysis. ROC curves were illustrated by IBM SPSS Statistics version 24.0.

## Results

### Significantly dysregulated expression of LINC01133, ZEB1-AS1 and ABHD11-AS1 in luminal BC tissues and cell lines

**Fig. 1:**
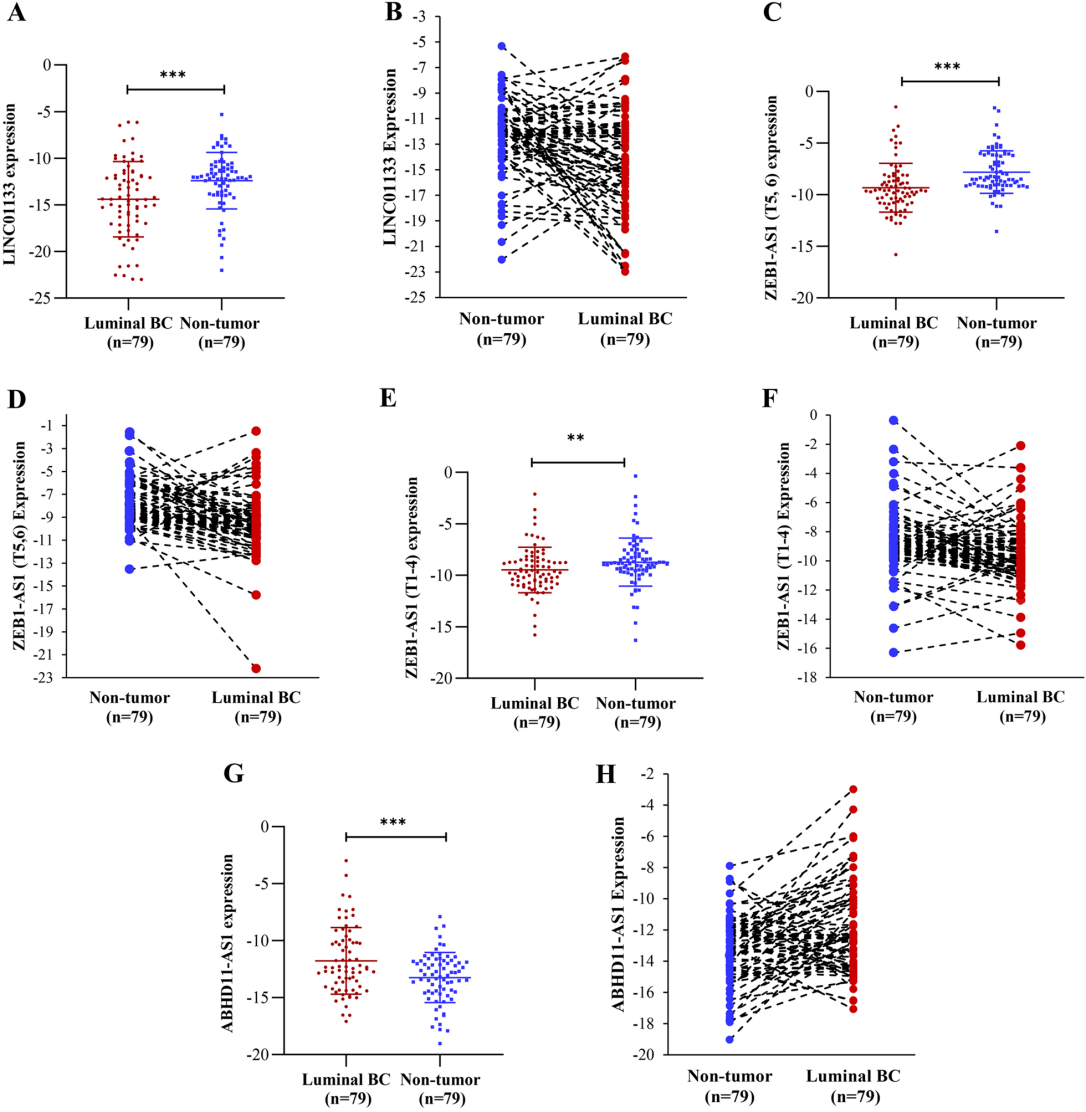
The expression level of LINC01133, ZEB1-AS1 and ABHD11-AS1 was altered in luminal BC tissues and cell lines compared to adjacent non-tumoral tissues and a normal breast cell line. The lncRNAs expression levels were detected by qRT-PCR in tumoral and non-tumoral tissues from 79 patients. **A**, **B** LINC01133 was significantly downregulated in luminal BC tissues (64% of cases). **C**, **D** ZEB1-AS1 (T5,6) was significantly downregulated in luminal BC tissues (80% of cases). **E**, **F** ZEB1-AS1 (T1-4) was significantly downregulated in luminal BC tissues (71% of cases). **G**, **H** ABHD11-AS1 was significantly upregulated in luminal BC tissues (70% of cases). All PCR reactions were performed three times and compared by paired sample t-test. B2M was used as an internal control. Data was presented as the mean ± SD. (***p < 0.001; **p < 0.01)

According to the results of the qRT-PCR assay, LINC01133 and ZEB1-AS1 were significantly downregulated in luminal A and B BC tissues (*p*_*LINC01133*_ = 0.001) (*p*_*ZEB1-AS1(T5,6)*_ < 0.001) (*p*_*ZEB1-AS1(T1-4)*_ = 0.002) (Fig. 1A, C, E). Also, ABHD11-AS1 expression was upregulated considerably in luminal BC tissues (*p* < 0.001, Fig. 1G). As shown in Fig. 1B, D, F), LINC01133, ZEB1-AS1(T5,6), and ZEB1-AS1(T1-4) were downregulated in 64%, 80% and 71% of samples, respectively. Moreover, ABHD11-AS1 was upregulated in 70% of cases (Fig. 1H).

**Fig. 2:**
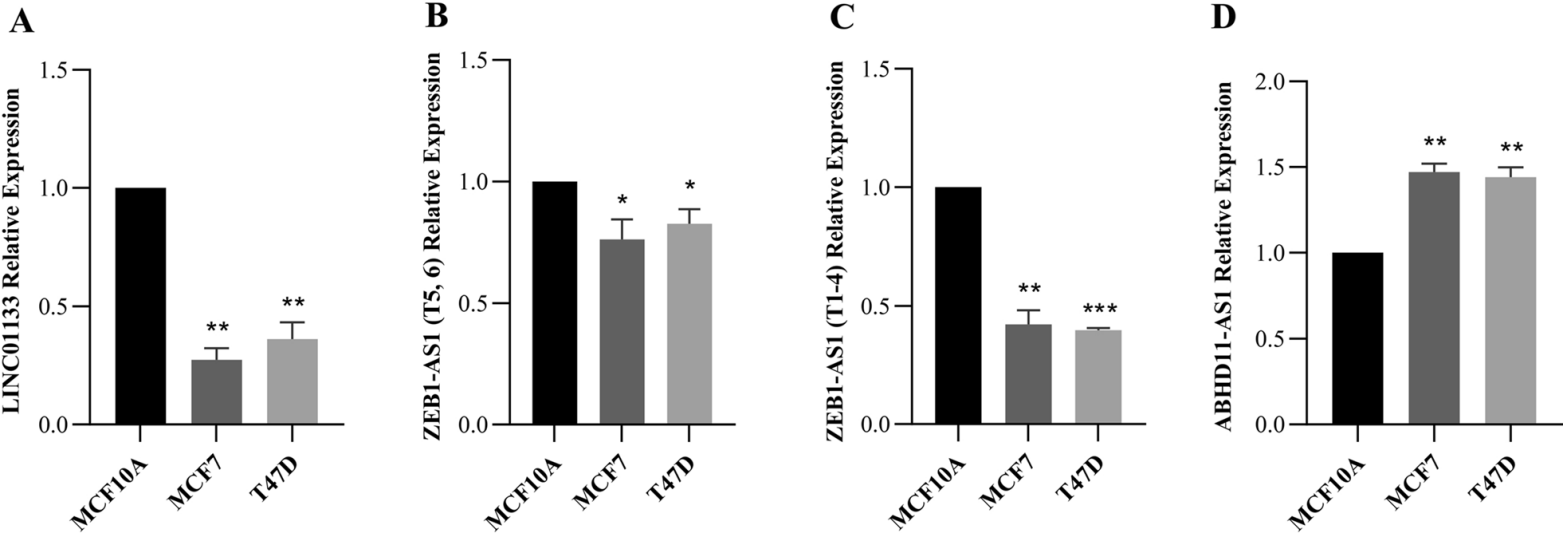
The expression level of LINC01133, ZEB1-AS1 and ABHD11-AS1 in luminal BC cell lines, T47D and MCF7, compared to a normal breast cell line (MCF10A). **A**–**C** LINC01133, ZEB1-AS1 (T5,6) and ZEB1-AS1 (T1-4) was significantly downregulated in luminal BC cell lines. **D** ABHD11-AS1 was considerably upregulated in luminal cell lines (***p < 0.001; **p < 0.01; *p < 0.05)

The results obtained from two luminal BC cell lines, T47D and MCF7, were consistent with the tissue expression data. The expression of LINC01133 and ZEB1-AS1 was decreased in the mentioned luminal cell lines compared with normal BC cell line (Fig. 2A–C). ABHD11-AS1 expression was upregulated significantly in luminal cell lines (Fig. 2D).

### Correlation between LINC01133, ZEB1-AS1, and ABHD11-AS1 expression levels and the clinicopathological features

As shown in Tables 3 and 4, no significant correlation was detected between LINC01133, ZEB1-AS1(T1-4), ZEB1-AS1(T5,6), and ABHD11-AS1 expression with clinicopathological features. Also, according to Kaplan–Meier analysis, the expression of the lncRNAs was not significantly associated with overall survival of the patients (Additional file 1).

**Table 3:** Correlation of LINC01133 and ABHD11-AS1 expression with clinicopathological features in luminal BC patients

Characteristics	No. of cases (total: 79)	LINC01133 expression level			ABHD11-AS1 expression level		
		Low N (%)	High N (%)	P-value	Low N (%)	High N (%)	P-value
Age at diagnosis (2 missing, n = 77)							
≤ 40	20	9 (25.0%)	11 (26.8%)	0.86	7 (19.4%)	13 (31.7%)	0.22
> 40	57	27 (75.0%)	30 (73.2%)		29 (80.6%)	28 (68.3%)	
Tumor size (1 missing, n = 78)							
< 2 cm	23	11 (29.7%)	12 (29.3%)	0.93	9 (23.1%)	14 (35.9%)	0.43
2–5 cm	41	20 (54.1%)	21 (51.2%)		23 (59.0%) 18 (46.2%)		
> 5 cm	14	6 (16.2%)	8 (19.5%)		7 (17.9%)	7 (17.9%)	
Tumor subtype (n = 79)							
Luminal A	65	32 (80.0%)	33 (84.6%)	0.59	34 (81.0%)	31 (83.8%)	0.74
Luminal B	14	8 (20.0%)	6 (15.4%)		8 (19.0%)	6 (16.2%)	
Grade (n = 79)							
1	11	7 (18%)	4 (10.0%)	0.42	5 (12.2%)	6 (15.8%)	0.87
2	54	24 (61.5%)	30 (75.0%)		29 (70.7%)	25 (65.8%)	
3	14	8 (20.5%)	6 (15.0%)		7 (17.1%)	7 (18.4%)	
Stage (2 missing, n = 77)							
I	6	2 (5.4%)	4 (10.0%)	0.62	1 (2.4%)	5 (13.9%)	0.16
II	42	22 (59.4%) 20 (50.0%)			23 (56.1%)	19 (52.8%)	
III	29	13 (35.2%)	16 (40.0%)		17 (41.5%)	12 (33.3%)	
Pathology of tumors (7 missing, n = 72)							
DCIS	3	2 (5.9%)	1 (2.6%)	0.80	1 (3.0%)	2 (5.1%)	0.85
LCIS	3	1 (2.9%)	2 (5.3%)		1 (3.0%)	2 (5.1%)	
IDC	57	28 (82.4%)	29 (76.3%)		28 (85.0%)	29 (74.4%)	
ILC	4	1 (2.9%)	3 (7.9%)		1 (3.0%)	3 (7.7%)	
Others	5	2 (5.9%)	3 (7.9%)		2 (6.0%)	3 (7.7%)	
Progesterone receptor (n = 79)							
Negative	10	4 (10.5%)	6 (14.6%)	0.58	4 (9.8%)	6 (15.8%)	0.42
Positive	69	34 (89.5%)	35 (85.4%)		37 (90.2%)	32 (84.2%)	
Estrogen receptor (n = 79)							
Negative	0	0 (0.0%)	0 (0.0%)	–	0 (0.0%)	0 (0.0%)	–
Positive	79	38 (100.0%) 41 (100.0%)			40 (100.0%)	39 (100.0%)	
HER2 (n = 79)							
Negative	70	32 (86.5%)	38 (90.5%)	0.58	37 (90.2%)	33 (86.8%)	0.63
Positive	9	5 (13.5%)	4 (9.5%)		4 (9.8%)	5 (13.2%)	

Stage grouping is based on American Joint Committee on Cancer (AJCC). Estrogen receptor (ER), progesterone receptor (PR), and Her2/neu classification is based on IHC results. The positive cut-off point is determined according to IHC guideline of American Society of Clinical Oncology (ASCO)

*DCIS* Ductal Carcinoma in Situ, *LCIS* Lobular Carcinoma in Situ, *IDC* Invasive Ductal Carcinoma, *ILC* Invasive Lobular Carcinoma

**Table 4:** Correlation of ZEB1-AS1(T1-4) and ZEB1-AS1(T5,6) expression with clinicopathological features in luminal BC patients

Characteristics	No. of cases (total: 79)	ZEB1-AS1(T1-4) expression level			ZEB1-AS1(T5,6) expression level		
		Low N (%)	High N (%)	P-value	Low N (%)	High N (%)	P-value
Age at diagnosis (2 missing, n = 77)							
≤ 40	20	12 (30.8%)	8 (21.1%)	0.33	12 (31.6%)	8 (20.5%)	0.26
> 40	57	27 (69.2%)	30 (78.9%)		26 (68.4%)	31 (79.5%)	
Tumor size (1 missing, n = 78)							
< 2 cm	23	11 (28.9%)	12 (30.0%)	0.15	10 (25.7%)	13 (33.3%)	0.25
2–5 cm	41	17 (44.8%)	24 (60.0%)		24 (61.5%)	17 (43.6%)	
> 5 cm	14	10 (26.3%)	4 (10.0%)		5 (12.8%)	9 (23.1%)	
Tumor subtype (n = 79)							
Luminal A	65	36 (90.0%)	29 (74.4%)	0.07	32 (80.0%)	33 (84.6%)	0.59
Luminal B	14	4 (10.0%)	10 (25.6%)		8 (20.0%)	6 (15.4%)	
Grade (n = 79)							
1	11	6 (15.0%)	5 (12.8%)	0.80	6 (15.0%)	5 (12.8%)	0.80
2	54	28 (70.0%)	26 (66.7%)		26 (65%)	28 (71.8%)	
3	14	6 (15.0%)	8 (20.5%)		8 (20.0%)	6 (15.4%)	
Stage (2 missing, n = 77)							
I	6	3 (7.9%)	3 (7.7%)	0.94	3 (7.7%)	3 (7.9%)	0.82
II	42	20 (52.6%)	22 (56.4%)		20 (51.3%)	22 (57.9%)	
III	29	15 (39.5%)	14 (35.9%)		16 (41.0%)	13 (34.2%)	
Pathology of tumors (7 missing, n = 72)							
DCIS	3	1 (2.6%)	2 (5.9%)	0.20	1 (2.8%)	2 (5.6%)	0.32
LCIS	3	3 (7.9%)	0 (0.0%)		0 (0.0%)	3 (8.3%)	
IDC	57	29 (76.3%)	28 (82.4%)		30 (83.3%)	27 (75.0%)	
ILC	4	1 (2.6%)	3 (8.8%)		3 (8.3%)	1 (2.8%)	
Others	5	4 (10.6%)	1 (2.9%)		2 (5.6%)	3 (8.3%)	
Progesterone receptor (n = 79)							
Negative	10	4 (10.0%)	6 (15.4%)	0.47	4 (10.0%)	6 (15.4%)	0.47
Positive	69	36 (90.0%)	33 (84.6%)		36 (90.0%)	33 (84.6%)	
Estrogen receptor (n = 79)							
Negative	0	0 (0.0%)	0 (0.0%)	–	0 (0.0%)	0 (0.0%)	–
Positive	79	40 (100.0%)	39 (100.0%)		40 (100.0%)	39 (100.0%)	
HER2 (n = 79)							
Negative	70	37 (92.5%)	33 (84.6%)	0.69	36 (90.0%)	34 (87.2%)	0.69
Positive	9	3 (7.5%)	6 (15.4%)		4 (10.0%)	5 (12.8%)	

Stage grouping is based on American Joint Committee on Cancer (AJCC). Estrogen receptor (ER), progesterone receptor (PR), and Her2/neu classification is based on IHC results. The positive cut-off point is determined according to IHC guideline of American Society of Clinical Oncology (ASCO)

*DCIS* Ductal Carcinoma in Situ; *LCIS* Lobular Carcinoma in Situ, *IDC* Invasive Ductal Carcinoma, *ILC* Invasive Lobular Carcinoma

### Evaluation of diagnostic value of LINC01133, ZEB1-AS1 and ABHD11-AS1 in luminal BC

**Fig. 3:**
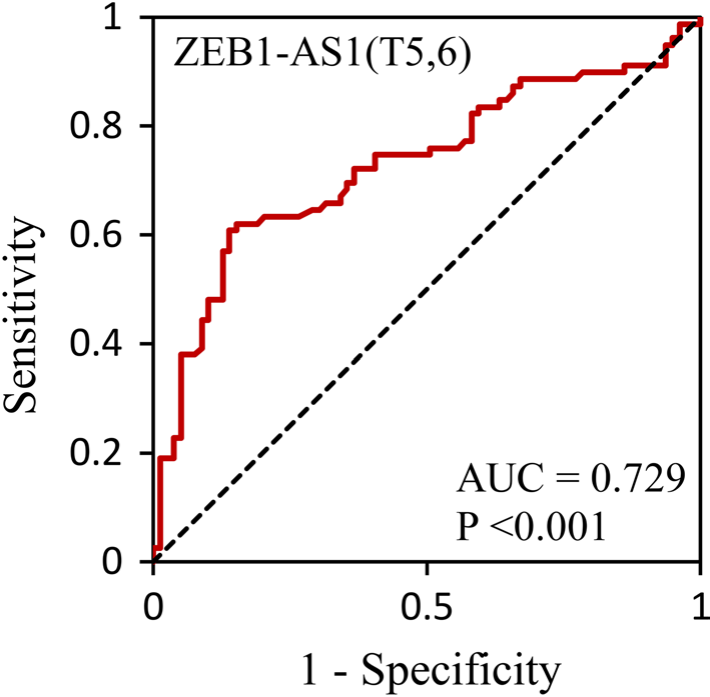
ROC curve of ZEB1-AS1(T5,6) in luminal BC. The curve was plotted to examine the diagnostic potential of ZEB1-AS1(T5,6) in discriminating luminal BC patients from healthy controls. The area under the curve (AUC) is 0.729 (*p* < 0.001)

The outcome of ROC analysis was defined based on the type of tissue (tumor or adjacent) and the predictor was characterized as gene expression level (upper or below the median expression). According to the ROC analysis of ZEB1-AS1(T5,6) shown in Fig. 3, the area under ROC curve is 0.729 across luminal A and B subtypes of BC (p_ZEB1-AS1(T5,6)_ < 0.001). On the other hand, LINC01133, ZEB1-AS1(T1-4), and ABHD11-AS1 were not suggested as good biomarkers for diagnosis of luminal subtypes of BC (*p*_*LINC01133*_ = 0.001/*AUC*_*LINC01133*_ = 0.661) (*p*_*ZEB1-AS1(T1-4)*_ = 0.005/*AUC*_*ZEB1-AS1(T1-4)*_ = 0.629) (*p*_*ABHD11-AS1*_ = 0.009*/AUC*_*ABHD11-AS1*_ = 0.375).

### Expression pattern of lncRNAs LINC01133, ZEB1-AS1 and ABHD11-AS1 among PAM50-breast cancer subtype classifications

According to the differential expression analysis, lower expression of LINC01133 was observed in luminal A and B BC subtypes*.* The expression of ZEB1-AS1 in luminal B and HER2 subtypes was lower than other groups. Moreover, significant differences of the expression of these lncRNAs were observed among different PAM50 subtypes (*p*_*LINC01133*_ = 1.13e−32) (*p*_ZEB1-AS1_ = 9.82e−5). On the other hand, luminal A and B had higher expression of ABHD11-AS1 followed by Normal-like, HER2, and Basal subtypes. The expression of ABHD11-AS1 was also significantly different among molecular subtypes of BC (*p*_ABHD11-AS1_ = 2.79e−8) (Additional file 2).

Besides, results obtained from the GENEVESTIGATOR database across luminal BC cell lines were consistent with the experimental data of the present study which indicated that LINC01133 and ZEB1-AS1 were downregulated in luminal cell lines. Also, expression analysis of these lncRNAs in several non-luminal BC cell lines showed that their expression was also downregulated across these cell lines (Additional files 3, 4). However, as ABHD11-AS1 is a novel lncRNA, no information was available about this lncRNA across GENEVESTIGATOR database.

### Genetic alterations of LINC01133, ZEB1-AS1 and ABHD11-AS1 across luminal BC

**Fig. 4:**
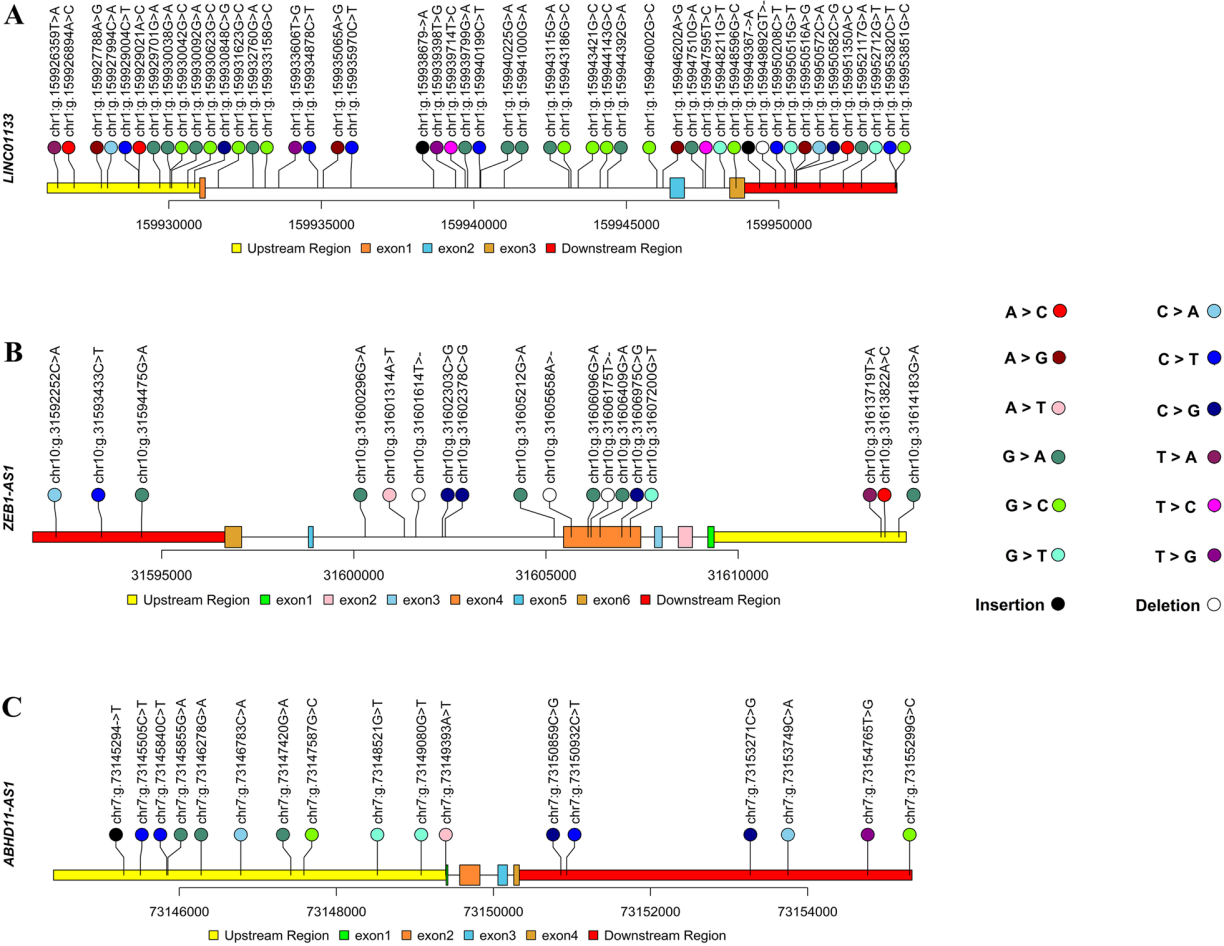
Genetic alterations within 5 kb upstream and downstream of the candidate lncRNAs across ER^+^ HER2^–^ BC, studied in ICGC. **A** LINC01133 mutations occur more frequently in intronic, downstream, and upstream regions, respectively. They are mostly of the substitution type. **B** ZEB1-AS1 mutations occur more frequently in intronic and exonic regions and are mostly of the substitution type. **C** ABHD11-AS1 mutations occur more frequently in upstream and downstream regions, respectively. ABHD11-AS1 mutations are also mostly of the substitution type. All of the data were obtained from ICGC and were illustrated by trackViewer R Package

According to the results obtained from the ICGC data portal, LINC01133 mutations in ER^+^ HER2^–^ BC take place most frequently in intronic, downstream, and upstream regions, respectively. Also, most LINC01133 mutations have been identified to be of the substitution type (Fig. 4A).

It was also found that ZEB1-AS1 mutations occur more frequently on introns and exons and are of the substitution type (Fig. 4B).

Most of ABHD11-AS1 mutations are substitutions which mostly take place in upstream and downstream regions (Fig. 4C).

### Co-expression gene network and functional annotation analysis

A list containing 420 luminal samples (230 luminal A and 190 luminal B) was obtained using UCSC Xena Browser and the Genomic Data Commons (GDC) databases (Additional file 5). Results obtained from the co-expression analysis of LINC01133, ZEB1-AS1, and ABHD11-AS1 across the selected luminal A and B BC samples suggested that these lncRNAs are significantly co-expressed with 256, 149 and 126 genes, respectively (*R* ≥ 0.4) (Additional file 6).

**Fig. 5:**
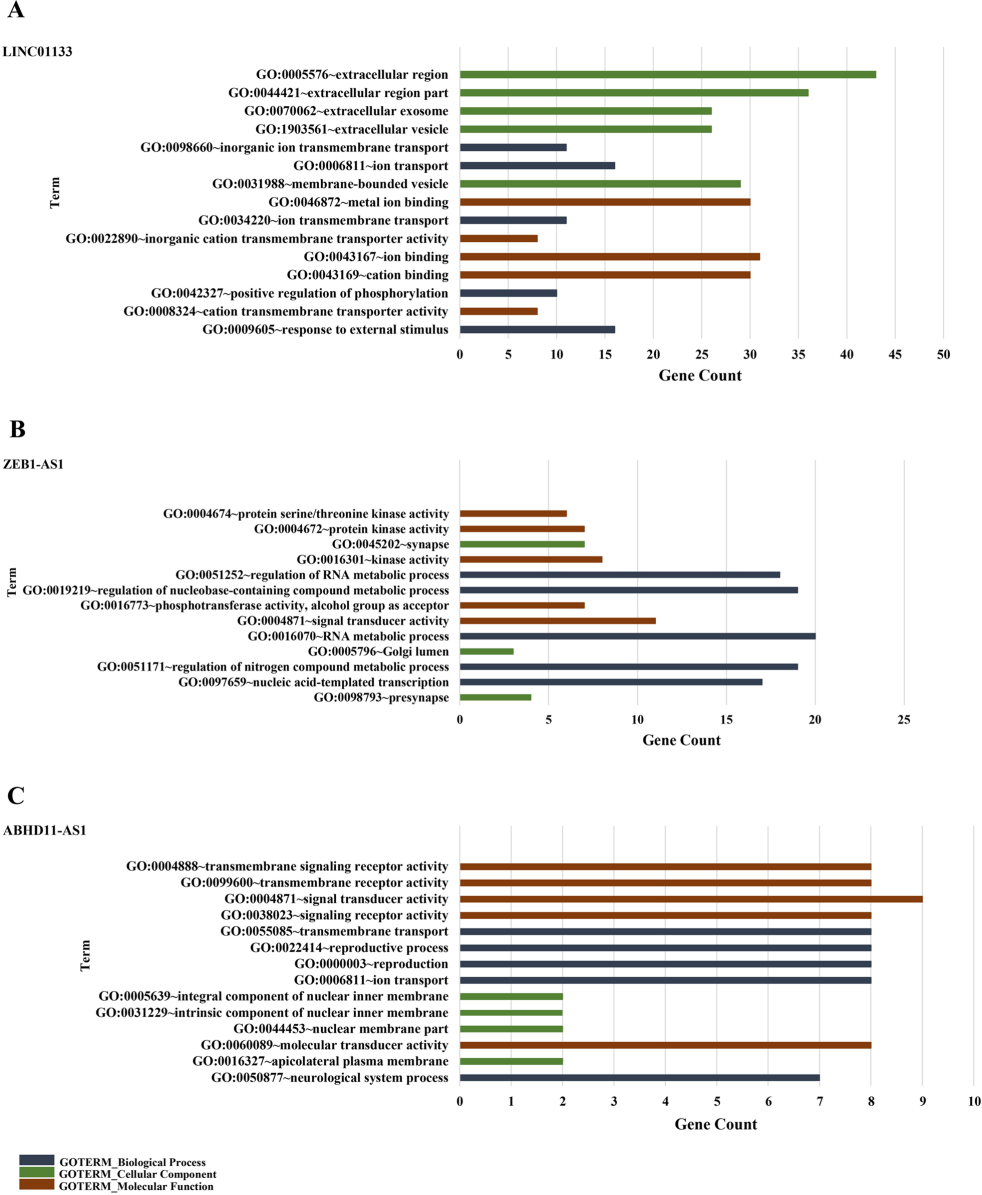
Top 5 gene ontology (GO) terms related to the co-expressed genes with the candidate lncRNAs for categories of biological process (BP), cellular component (CC), and molecular function (MF). **A** Bar chart of the GO term enrichment analysis of the co-expressed genes with LINC01133. **B** Bar chart of the GO term enrichment analysis of the co-expressed genes with ZEB1-AS1. **C** Bar chart of the GO term enrichment analysis of the co-expressed genes with ABHD11-AS1. The top GO terms were selected based on the amount of gene count and the chart was illustrated according to the data obtained from enrichment analysis. The columns were sorted from top to bottom according to significancy of the terms (*p* < 0.05). All of the data was obtained from DAVID database

Data retrieved from GO term enrichment analysis by DAVID included a list for categories of biological process (BP), cellular component (CC), and molecular function (MF) (Additional file 7). Also, results of summarizing and excluding redundant GO terms of the target lncRNAs of the present study, which were performed by REVIGO, were concordant with the data obtained from DAVID GO term enrichment analysis. The top 5 BP, CC, and MF terms related to each lncRNA are shown in Fig. 5. According to it, the genes co-expressed with LINC01133 are significantly involved in biological processes such as ion transport, response to external stimulus, ion transmembrane transport, positive regulation of phosphorylation, etc. It was also determined that molecular function of these genes considerably consists of ion/cation/metal binding, cation transmembrane transporter activity, etc. Each of the above activities takes place inside and/or in the vicinity of a cell. According to CC terms, it was predicted that the co-expressed genes with LINC01133 are considerably found in extracellular region/exosome, membrane-bounded vesicle and so on (*p* < 0.05) (Fig. 5A).

It has been found that the co-expressed genes with ZEB1-AS1 are considerably involved in the regulation of RNA metabolic process, regulation of nucleobase-containing compound metabolic process, nucleic acid-templated transcription, etc. As shown in Fig. 5B, the most significant molecular functions of these genes are signal transducer activity, kinase activity, phosphotransferase activity, etc. These genes are mostly enriched in the synapse and Golgi lumen (*p* < 0.05).

Moreover, the genes co-expressed with ABHD11-AS1 are considerably involved in biological processes such as transmembrane transport, reproductive process, ion transport, neurological system process, etc. They are also considerably involved in various molecular functions including signal transducer activity, transmembrane signaling receptor activity, molecular transducer activity, etc. Additionally, the GO cellular component terms suggested that the co-expressed genes with ABHD11-AS1 are considerably enriched in the intrinsic component of nuclear membrane part, apicolateral plasma membrane, etc. (*p* < 0.05) (Fig. 5C).

**Fig. 6:**
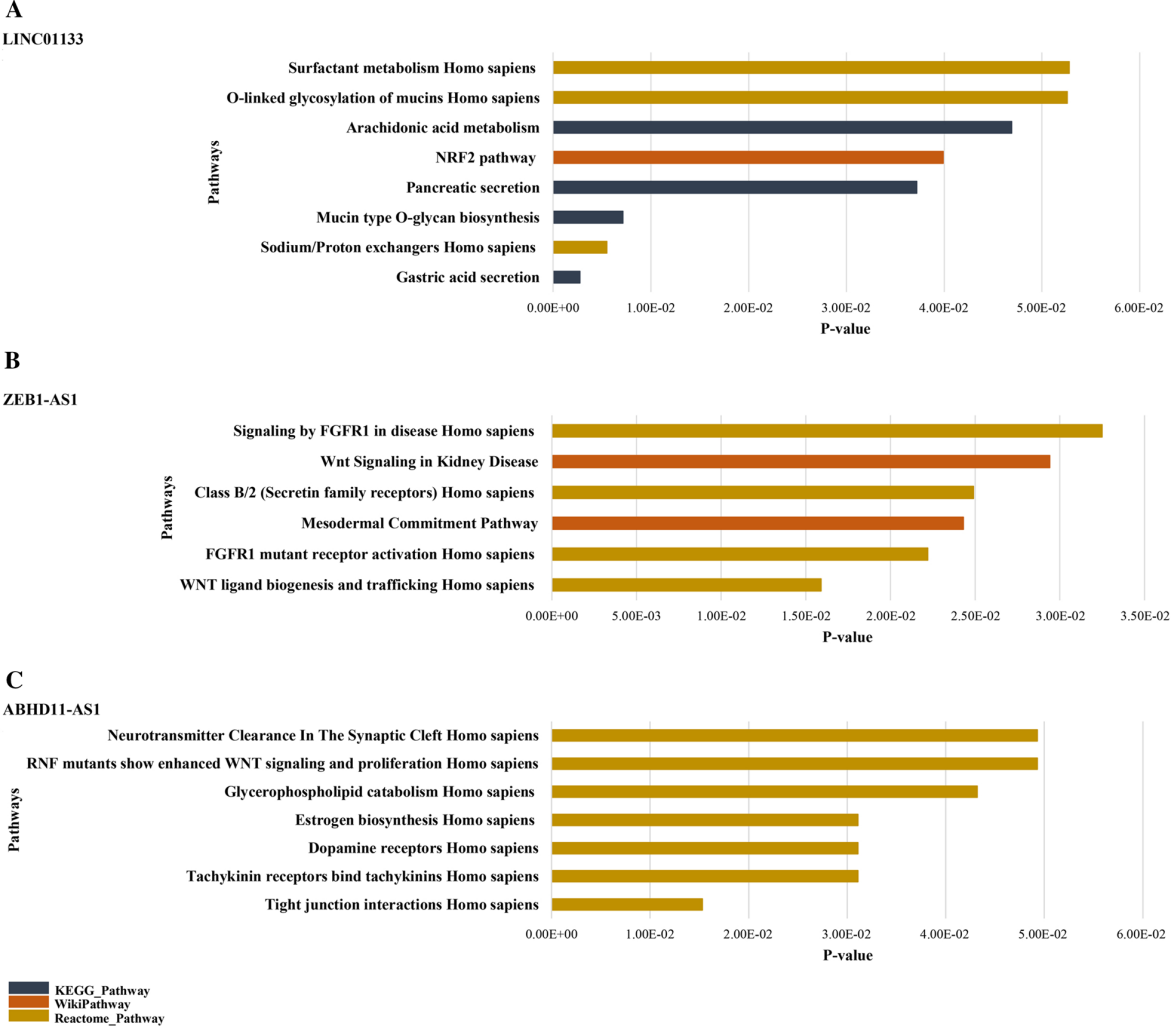
The pathway analysis of the hub genes showed involvement of the co-expressed genes with the candidate lncRNAs in cancer-related pathways. **A** Bar chart of the KEGG, Reactome and WikiPathways enrichment analysis of the co-expressed genes with LINC01133. **B** The Reactome pathway enrichment analysis of the co-expressed genes with ZEB1-AS1. **C** The Reactome and WikiPathways enrichment analysis of the co-expressed genes with ABHD11-AS1. The columns were sorted from top to bottom according to significancy of the terms (*p* < 0.05). All of the data was obtained from Enrichr web server

The results of gene set enrichment analysis by Enrichr are demonstrated in Fig. 6. According to KEGG, Reactome and WikiPathways analysis, the co-expressed genes with LINC01133 are mainly involved in sodium/proton exchanges, mucin-type O-glycan biosynthesis, and nuclear factor erythroid 2-related factor 2 (NRF2) pathway (Fig. 6A). Moreover, ZEB1-AS1 co-expressed genes are significantly enriched in tight junction interactions, estrogen biosynthesis, glycerophospholipid catabolism, and Wnt signaling pathway across luminal BC (Fig. 6B). The co-expressed genes with ABHD11-AS1 are mainly involved in the processes of Wnt ligand biogenesis, FGFR1 mutant receptor activation, mesodermal commitment pathway, and Wnt signaling (Fig. 6C).

**Fig. 7:**
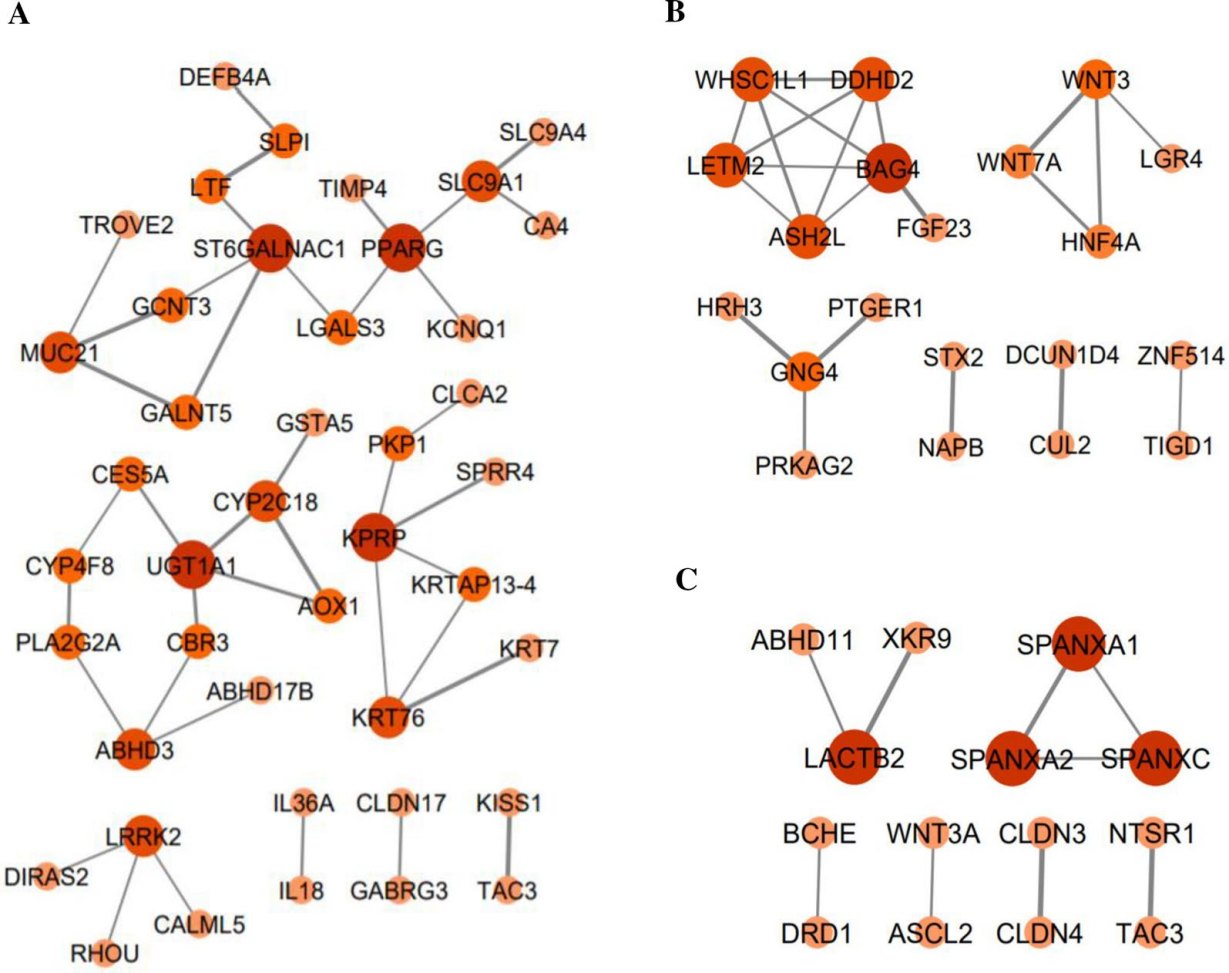
The analyzed protein–protein networks of the co-expressed genes with the target lncRNAs confirmed strong interactions between a number of genes. **A** 42 genes co-expressed with LINC01133 have strong interactions with each other. **B** 20 genes co-expressed with ZEB1-AS1 have strong interactions with each other. **C** 14 genes co-expressed with ABHD11-AS1 have strong interactions with each other. The thicker edge and the stronger node color are representative of the higher STRING combined-score and the higher degree, respectively. Data was obtained from the STRING database and was illustrated by Cytoscape (*interaction score* > 0.4)

Finally, the association analysis of the genes co-expressed with LINC01133 demonstrated that 42 out of 256 genes had a strong PPI (*interaction score* > 0.4) with each other (Fig. 7A). Also, according to PPI network analysis of the co-expressed genes with ZEB1-AS1 and ABHD11-AS1, 20 and 14 genes have been recognized with strong interactions with each other, respectively (Fig. 7B, C).

According to the CytoHubba, the genes KPRP, PPARG, ST6GALNAC1, and UGT1A1 were adopted as the top hub genes of the PPI network of LINC01133 co-expressed genes ranked by degree metric. Also, five (BAG4, ASH2L, DDHD2, LETM2, and WHSC1L1) and four (LACTB2, SPANXA1, SPANXA2, and SPANXC) genes were adopted as the hob genes related to ZEB1-AS1 and ABHD11-AS1 networks, respectively.

## Discussion

The luminal subtype of BC, which has shown better prognosis among the other subtypes, accounts for approximately two-thirds of this cancer [43, 44]. However, it is reported that the luminal B subtype has demonstrated poorer recurrence-free survival in adjuvant treatment categories in comparison to luminal A [44]. Also, it was proved that a large percentage of ER^+^ patients with lymph node positive benefit less from adjuvant chemotherapy [43]. Due to the heterogeneity of this cancer, the same therapies may demonstrate diverse outcomes in patients. So, distinguishing the underlying mechanisms and specific biomarkers can help the individualized treatment of especially high-risked luminal patients.

In this study, LINC01133 and ZEB1-AS1 were significantly downregulated in luminal A and B BC tissues compared to their adjacent non-tumoral tissues. Also, they were downregulated in luminal cell lines, namely T47D and MCF7, compared to a normal breast cell line (MCF10A). Moreover, ABHD11-AS1 was upregulated considerably in the mentioned tissues and cell lines.

LncRNAs have shown greater expression variability among different individuals and a greater degree of tissue-specificity, compared with coding-genes [45]. The downregulation of LINC01133, ZEB1-AS1(T1-4), and ZEB1-AS1(T5,6) was detected in 64%, 71% and 80% of patients, respectively. Also, the upregulation of ABHD11-AS1 was found in a large proportion of patients (70% of cases). Song et al. reported that the downregulation of LINC01133 is considerably associated with the poor prognosis of BC patients [46]. On the other hand, Luo et al. suggested that ZEB1‐AS1 promotes triple-negative breast cancer progression [47]. So, this lncRNA may also play an important role in luminal BC patients, although further investigation is needed. Also, ZEB1-AS1(T5,6) may be used as a biomarker for luminal BC progression. However, more in-vitro tests are needed for more accurate conclusions.

Limited studies have been performed on the dysregulation of these lncRNAs and their roles in cancers so far. So, the combination of various datasets as well as the analysis of their co-expressed genes could provide better identification of functional roles of the mentioned lncRNAs in luminal BC. According to the result of TCGA data analysis available in TANRIC database, LINC01133, ZEB1-AS1, and ABHD11-AS1 expression were significantly different among PAM50 subtype classification. Besides, the expression pattern of LINC01133 and ZEB1-AS1 across luminal and non-luminal BC cell lines was consistent with the data from TANRIC database and also experimental data of the present study. According to the data obtained from the ICGC data portal, most of the mutations of LINC01133, ZEB1-AS1, and ABHD11-AS1 in ER^+^ HER2^–^ patients are from substitution category. The effect of mutations and variations of lncRNAs have not been extensively studied as they constitute a novel class of RNAs. Ponjavic et al. demonstrated that functional lncRNAs show reduced single substitutions, deletions and insertions in their sequences. Although nucleotide substitutions occur mostly in protein-coding sequences compared with noncoding sequences, their effect on lncRNAs are so significant and should not be overlooked [48]. Substitution mutations in lncRNAs may contribute to the pathogenesis of various cancers by disrupting the secondary structure of RNAs [18, 49]. The RNA secondary structure plays important roles in multiple cellular processes such as gene regulation and localization, splicing, stability and also translation [50]. Also, mutations in the upstream regions of lncRNAs, which constitute a considerable percentage of LINC01133 and ABHD11-AS1 mutations, can have significant effects on them. For example, the presence of single-nucleotide polymorphisms in the promoter of a lncRNA may have an effect on its expression pattern [49]. So, further studies are recommended to determine the effect of these mutations on the structure of the mentioned lncRNAs.

Remarkably, the protein-coding genes are significantly better annotated than lncRNAs [51]. So, in this study, an attempt was made to determine the function of the target lncRNAs with the help of the known functional information of some coding and non-coding genes, using “guilt-by-association” principle. According to this principle, genes that are involved in some related and/or similar biological pathways may show similar expression patterns across different experimental conditions [52].

According to the “guilt-by-association” principle and the GO term enrichment analysis of the co-expressed genes with LINC01133, this lncRNA might be involved in ion transport, response to external stimulus, and positive regulation of phosphorylation, all of which are cancer‐associated biological processes. Also, ion/cation/metal binding and cation transmembrane transporter activity are known as the most crucial molecular functions in which LINC01133 is involved. LINC01133 might be mostly found in extracellular region/exosome and membrane-bounded vesicles.

According to the results obtained from the GO term enrichment analysis of ZEB1-AS1 co-expressed genes, this lncRNA is involved in the regulation of RNA metabolic process, regulation of nucleobase-containing compound metabolic process, nucleic acid-templated transcription, etc. Deregulation of all of these biological processes can lead to the development and progression of various cancers, including BC. In addition, ZEB1-AS1 might be plausibly involved in molecular functions including signal transducer activity, kinase activity and phosphotransferase activity, all of which can be linked to cancer progression. ZEB1-AS1 might be mostly enriched in synapse and Golgi lumen.

ABHD11-AS1 might be involved in transmembrane transport, reproductive process, ion transport, neurological system process, etc. ABHD11-AS1 also might be considerably involved in different molecular functions, like signal transducer activity, transmembrane signaling receptor activity, molecular transducer activity, etc. Dysregulation of all of the mentioned functions can lead to the development of various cancers. Moreover, ABHD11-AS1 is likely to be found in intrinsic component of nuclear membrane part, apicolateral plasma membrane, etc.

Significantly enriched KEGG, Reactome and WikiPathways of the co-expressed genes with LINC01133 include sodium/proton exchanges, mucin-type O-glycan biosynthesis, and NRF2 pathway. Abnormal glycoprotein structure of tumor cells can affect the survival, growth and metastasis of cancer cells [53]. Nuclear receptors (NR) are known as regulators of physiological processes and play oncogenic or anti-oncogenic roles in cancerous cells [54]. Growing evidence support the involvement of several NRs in the regulation of various pathways related to the initiation and development of BC [55]. Overexpression of Nrf2 has been shown to increase the expression of glucose‐6‐phosphate dehydrogenase (G6PD) and Hypoxia‐inducing factor 1α (HIF‐1α) in BC cell lines, including MCF7. Also, overexpression of Nrf2 increases Notch1 expression via the G6PD/HIF-1α pathway. Notch signaling pathway regulates BC cell proliferation and migration by affecting the downstream gene, HES‐1, and the epithelial-to-mesenchymal transition (EMT) pathway, respectively [56]. All of these evidences suggest that LINC01133 could play an essential role in BC.

Moreover, according to the pathway enrichment analysis, ZEB1-AS1 might be involved in tight junction interactions, estrogen biosynthesis, glycerophospholipid catabolism, and Wnt signaling pathway. All of these pathways can be associated with the progression and development of BC. Steroid hormones increase cell proliferation in BC [57]. Also, alterations in tight junction complexes could facilitate BC initiation and progression by impairing their control over crucial cellular processes such as cell motility and polarity [58]. Moreover, the Wnt pathway is significantly activated in breast tumors [59].

ABHD11-AS1 might also be mainly involved in Wnt ligand biogenesis, FGFR1 mutant receptor activation, mesodermal commitment pathway, and Wnt signaling pathway. The involvement of fibroblast growth factors (FGF) has been found in various cellular processes, including proliferation, drug resistance, anti-apoptosis and angiogenesis. Also, amplification of FGFR1 has been found in ER^+^ BC [60].

Data from STRING database showed that 42, 20, and 14 co-expressed genes with LINC01133, ZEB1-AS1 and ABHD11-AS1 have strong interactions with each other. These data confirmed the involvement of these lncRNAs and their co-expressed genes in similar biological pathways.

Taken together, the dysregulation of the three potential lncRNAs, LINC01133, ZEB1-AS1, and ABHD11-AS1 across luminal A and B subtypes of BC was reported in the present study. The bioinformatics analyses performed in this study helped us better identify the possible role of these lncRNAs in luminal BC. However, more experimental studies are needed to confirm these findings and to verify the exact roles of the mentioned lncRNAs.

## Conclusions

All our findings have demonstrated the downregulation of LINC01133 and ZEB1-AS1 across luminal BC tissue specimens and cell lines compared to adjacent non-tumoral tissues and cell line. On the other hand, ABHD11-AS1 was upregulated in the mentioned BC tissues and cell lines.

## Supplementary Information

**Additional file 1. Figure S1.** The kaplan-meier survival functions of LINC01133, ZEB1-AS1(T1-4), ZEB1-AS1(T5,6), and ABHD11-AS1 across luminal BC samples.

**Additional file 2. Figure S2.** Differential expression of LINC01133, ZEB1-AS1 and ABHD11-AS1 across different subtypes of BC, obtained by TANRIC database.

**Additional file 3. Figure S3.** The expression level of LINC01133 across various BC cell lines, obtained by GENEVESTIGATOR database.

**Additional file 4. Figure S4.** The expression level of ZEB1-AS1 across various BC cell lines, obtained by GENEVESTIGATOR database.

**Additional file 5. File S1.** The list of 420 luminal samples obtained from UCSC Xena Browser and the Genomic Data Commons (GDC) database.

**Additional file 6. File S2.** The list of co-expressed genes with LINC01133, ZEB1-AS1 and ABHD11-AS1 across the selected luminal A and B BC samples.

**Additional file 7. File S3.** The list of biological process (BP), cellular component (CC), and molecular function (MF) categories, retrieved from DAVID database.

## Data Availability

The datasets supporting the conclusions of this article are available in: [Lnc2Cancer database] at (https://www.bio-bigdata.com/lnc2cancer/). [UCSC Xena browser] at (http://xena.ucsc.edu/); [GDC database] at (https://portal.gdc.cancer.gov/). [TANRIC database] at (https://www.tanric.org); [GENEVESTIGATOR database] at (https://genevestigator.com); [International Cancer Genome Consortium] at (https://dcc.icgc.org). [DAVID database] at (https://david.ncifcrf.gov). [REVIGO database] at (https://revigo.irb.hr). [Cytoscape software] at (https://www.cytoscape.org). [STRING database] at (https://string-db.org). [Enrichr database] at (https://maayanlab.cloud/Enrichr/). Citation of all data is provided in the references list. The datasets supporting the conclusions of this article are also included within the article and its additional files.

## Abbreviations

BC: Breast cancer
LncRNAs: Long non-coding RNAs
qRT-PCR: Quantitative real-time polymerase chain reaction
KEGG: Kyoto Encyclopedia of Genes and Genomes
ER: Estrogen receptor
PR: Progesterone receptor
HER2: Human epidermal growth factor receptor 2
BCRC-BB: Breast Cancer Research Biobank
TUMS: Tehran University of Medical Sciences
EGF: Epidermal growth factor
cDNA: Complementary DNA
β2M: Beta-2-Microglobulin
PCR: Polymerase chain reaction
GDC: Genomic Data Commons
BRCA: Breast invasive carcinoma
ICGC: The International Cancer Genome Consortium
DAVID: The Database for Annotation, Visualization and Integrated Discovery
GO: Gene ontology
PPI: Protein–protein interaction
IBM SPSS Statistics: Statistical Package for the Social Sciences
SD: Standard deviation
ROC: Receiver operating characteristics
ICGC: The International Cancer Genome Consortium
BP: Biological process
CC: Cellular component
MF: Molecular function
NRF2: Nuclear factor erythroid 2-related factor 2
NR: Nuclear receptors
G6PD: Glucose‐6‐phosphate dehydrogenase
EMT: Epithelial-to-mesenchymal transition
HIF‐1α: Hypoxia‐inducing factor 1α
FGF: Fibroblast growth factors
