# Supplementary figures and images for: Expression profiles and functional prediction of long non-coding RNAs LINC01133, ZEB1-AS1 and ABHD11-AS1 in the luminal subtype of breast cancer
Source: J Transl Med. 2021 Aug 26;19:364. doi: 10.1186/s12967-021-03026-7 (PMC8390237; doi:10.1186/s12967-021-03026-7)

Kaplan-meier Survival Functions

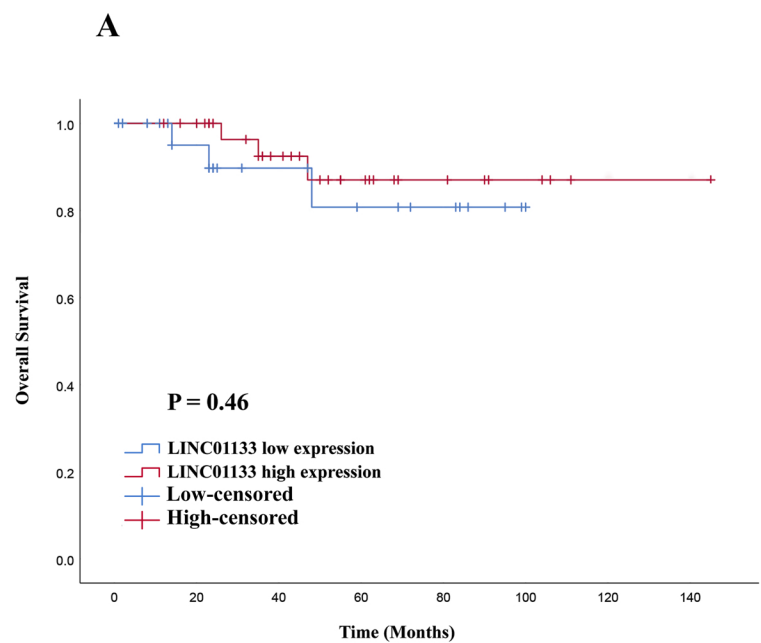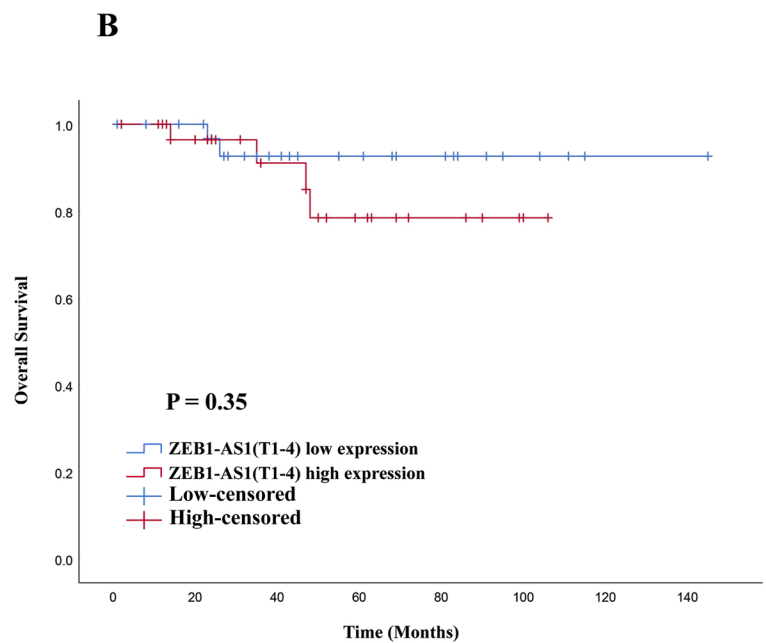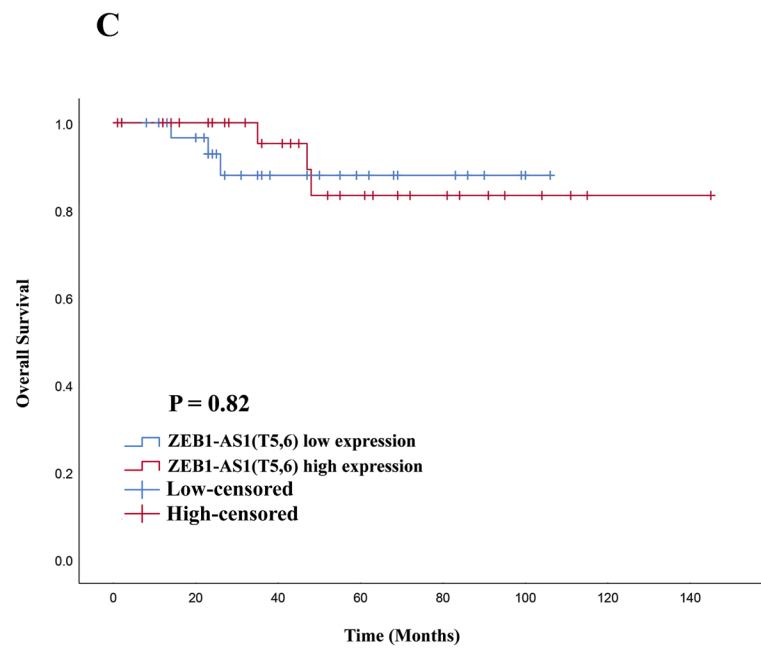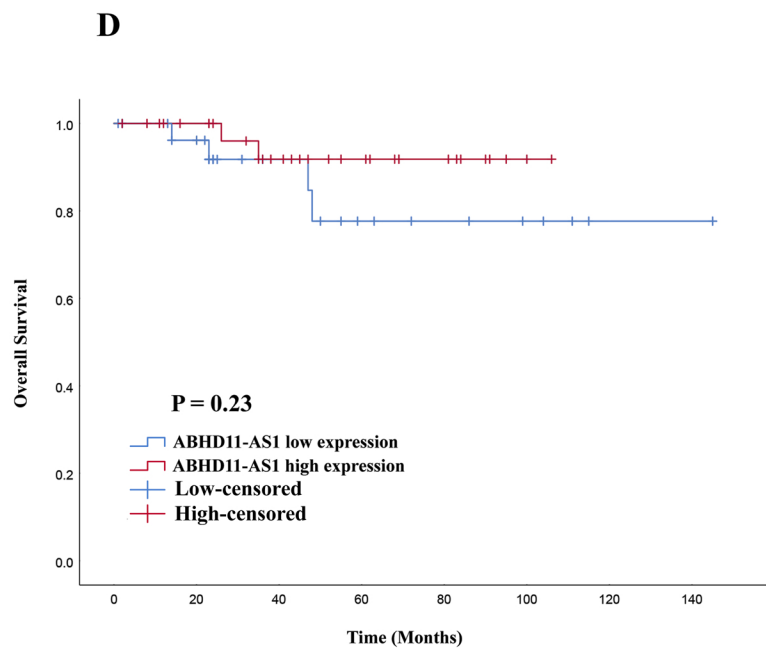

Supplement: Supplementary file 1 — Additional file 1. Figure S1. The kaplan-meier survival functions of LINC01133, ZEB1-AS1(T1-4), ZEB1-AS1(T5,6), and ABHD11-AS1 across luminal BC samples. [file 12967_2021_3026_MOESM1_ESM.pdf]

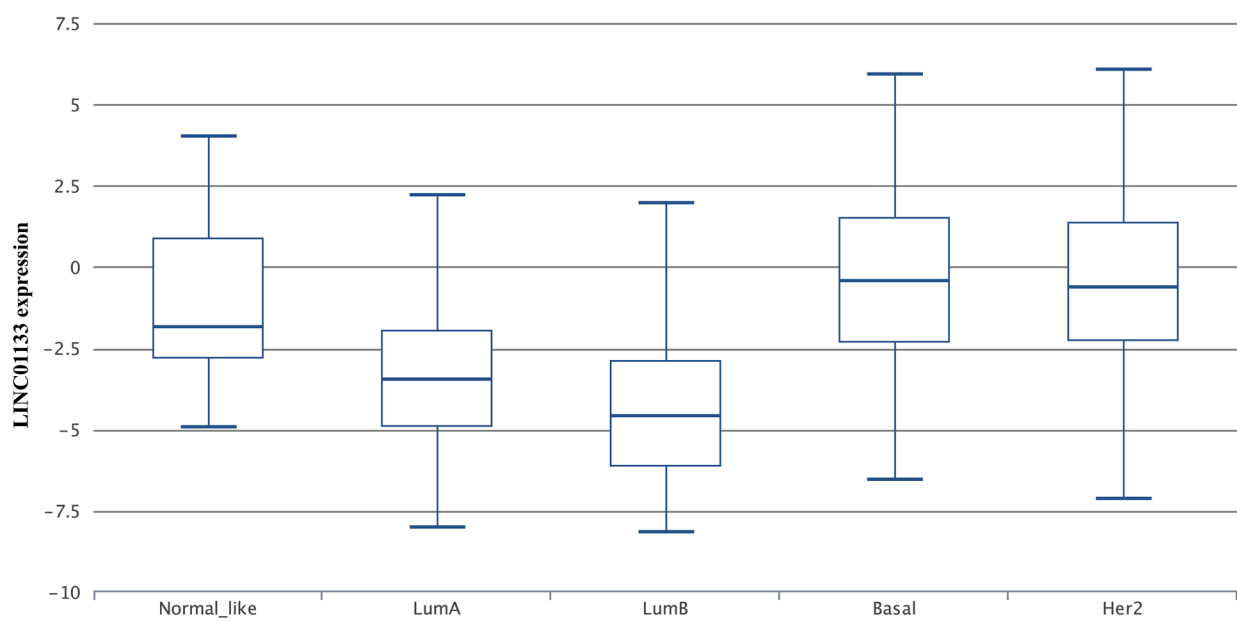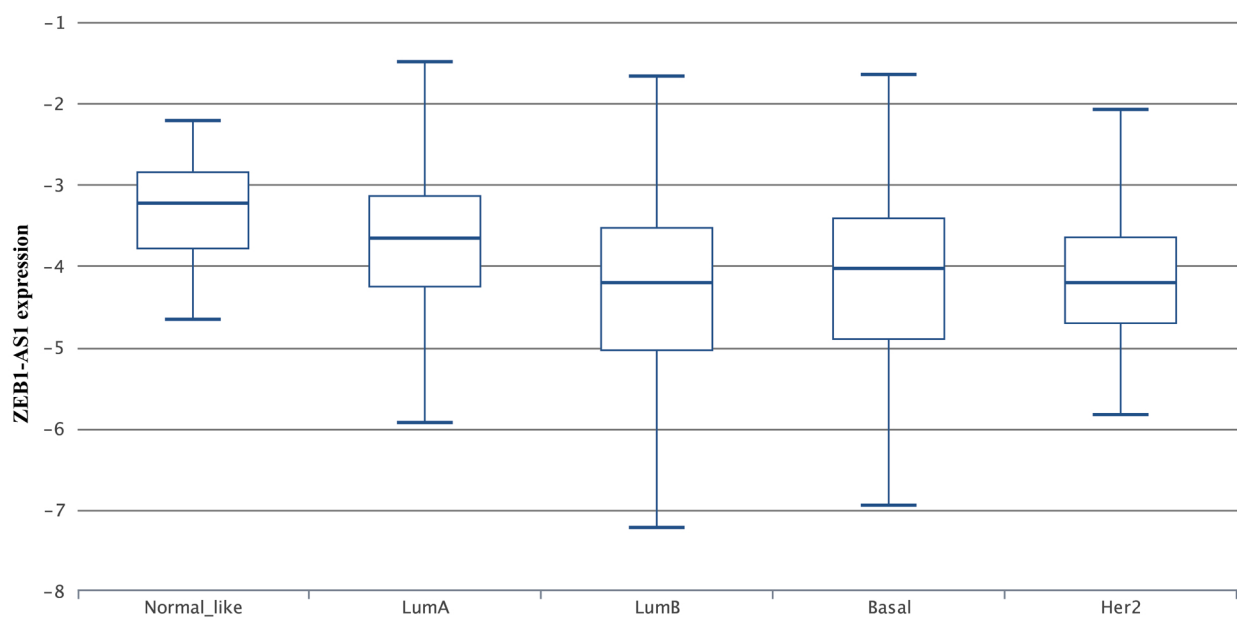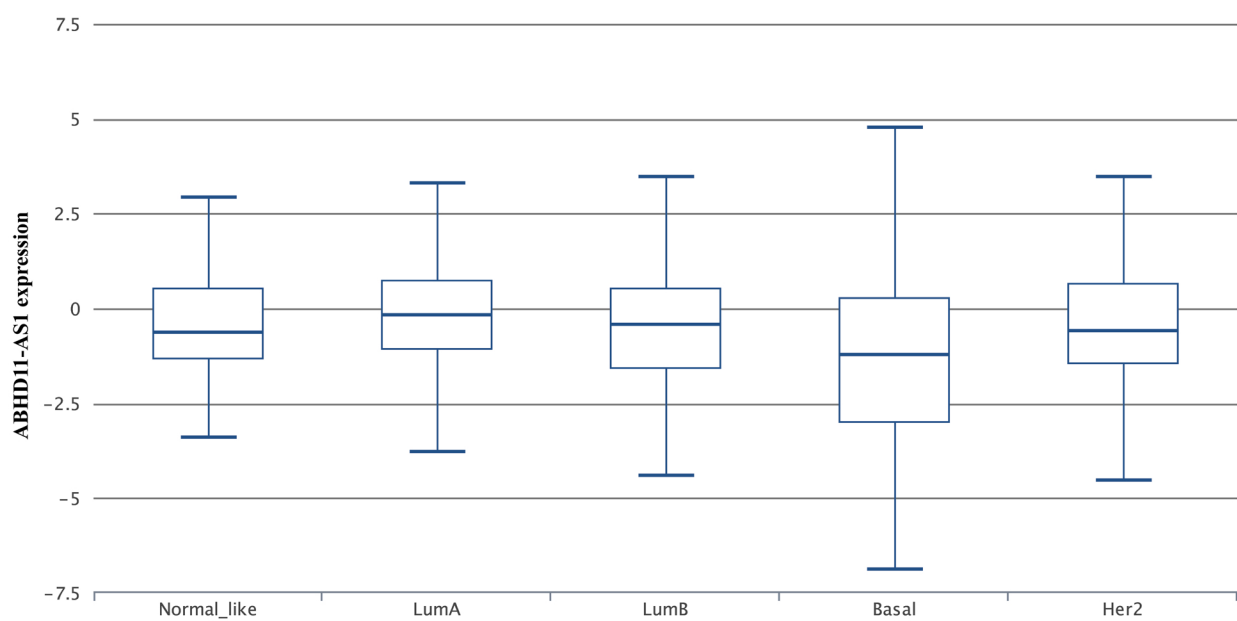

Supplement: Supplementary file 2 — Additional file 2. Figure S2. Differential expression of LINC01133, ZEB1-AS1 and ABHD11-AS1 across different subtypes of BC, obtained by TANRIC database. [file 12967_2021_3026_MOESM2_ESM.pdf]

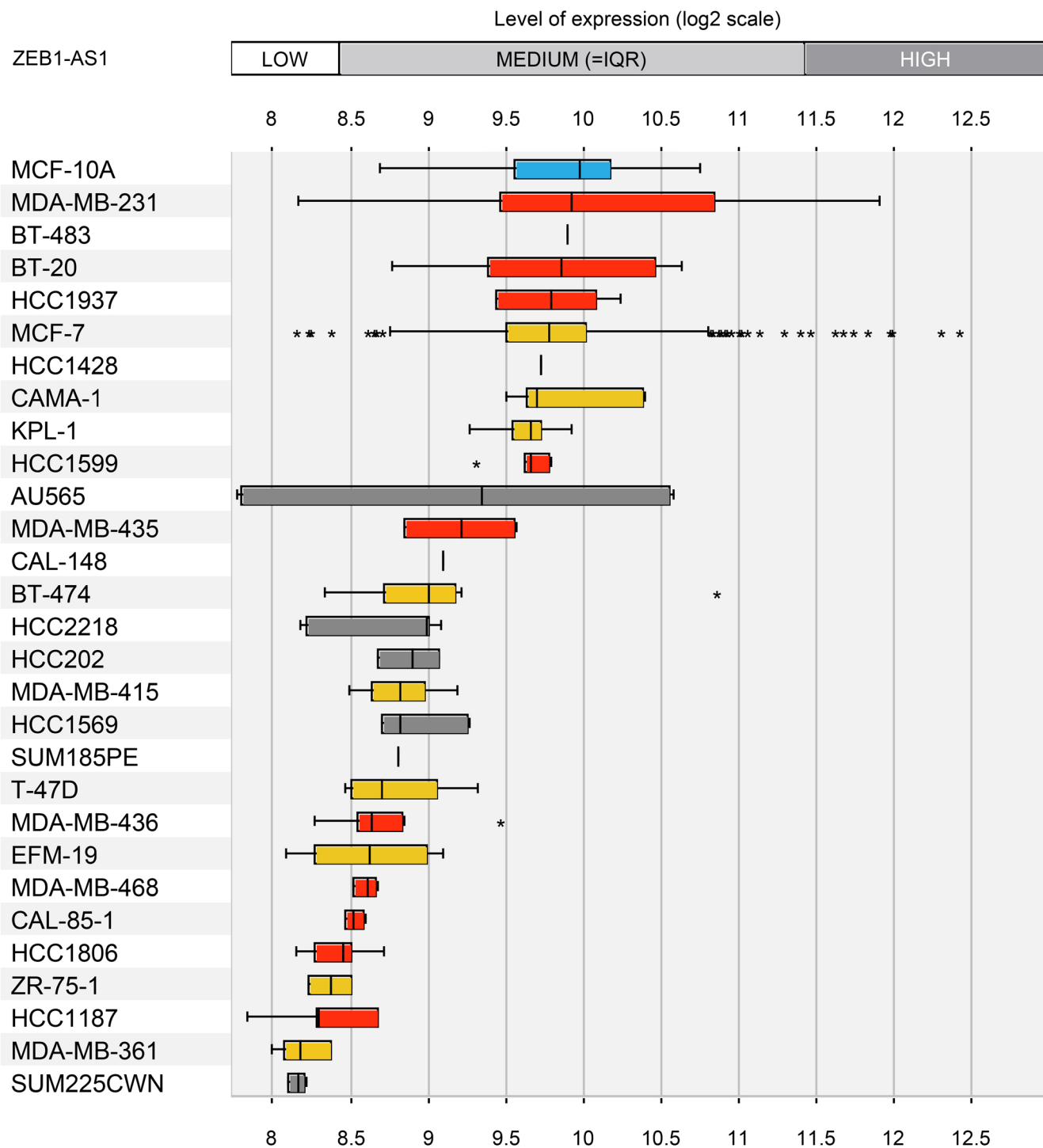

Supplement: Supplementary file 4 — Additional file 4. Figure S4. The expression level of ZEB1-AS1 across various BC cell lines, obtained by GENEVESTIGATOR database. [file 12967_2021_3026_MOESM4_ESM.pdf]
