# Supplementary material for: Expression profiles and functional prediction of long non-coding RNAs LINC01133, ZEB1-AS1 and ABHD11-AS1 in the luminal subtype of breast cancer
Source: J Transl Med. 2021 Aug 26;19:364. doi: 10.1186/s12967-021-03026-7 (PMC8390237; doi:10.1186/s12967-021-03026-7)
Supplement: Supplementary file 3 — Additional file 3. Figure S3. The expression level of LINC01133 across various BC cell lines, obtained by GENEVESTIGATOR database. [file 12967_2021_3026_MOESM3_ESM.pdf]

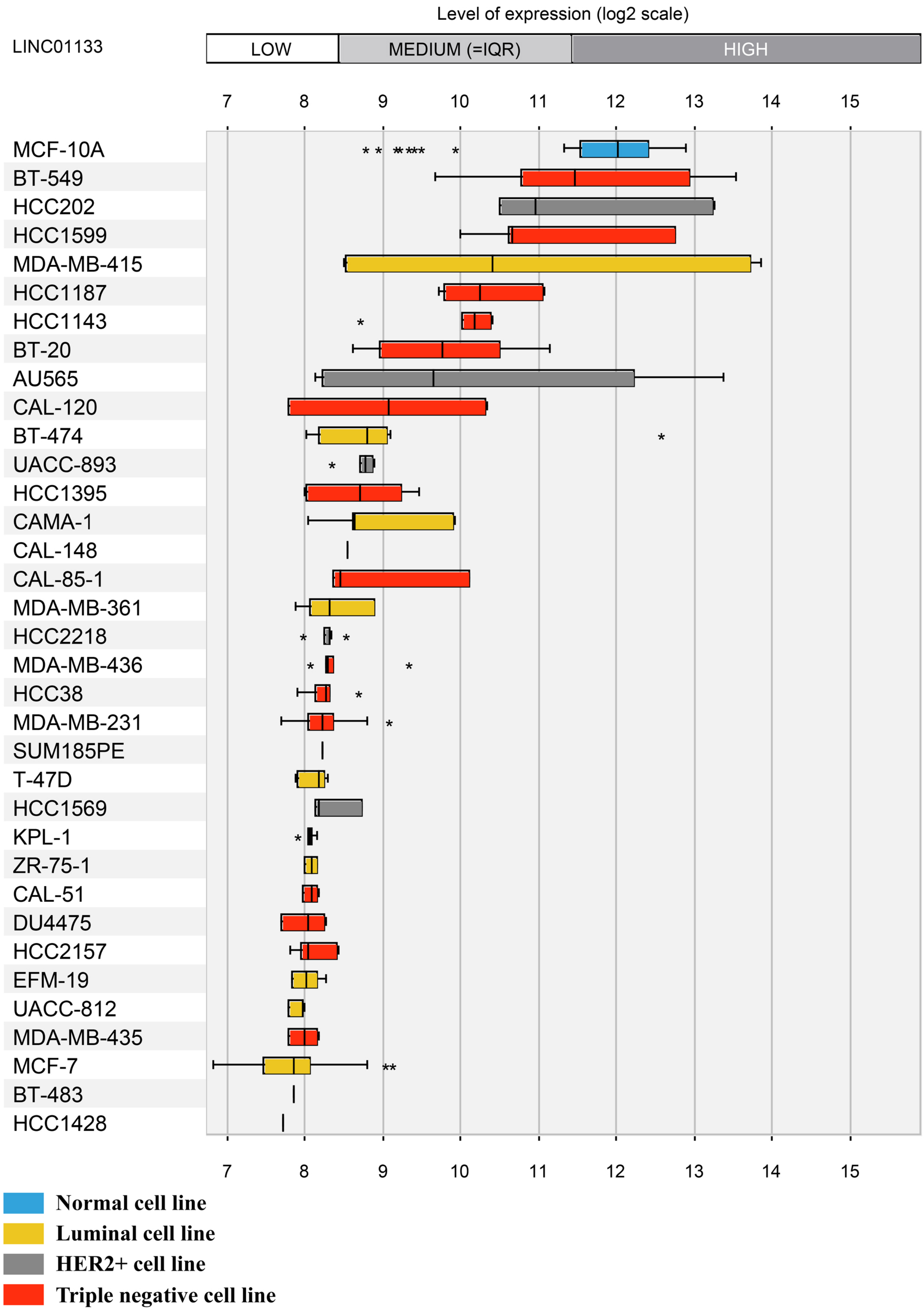

LINC01133
Level of expression (log2 scale)
LOW
MEDIUM (=IQR)
HIGH
7
8
9
10
11
12
13
14
15
MCF-10A
BT-549
HCC202
HCC1599
MDA-MB-415
HCC1187
HCC1143
BT-20
AU565
CAL-120
BT-474
UACC-893
HCC1395
CAMA-1
CAL-148
CAL-85-1
MDA-MB-361
HCC2218
MDA-MB-436
HCC38
MDA-MB-231
SUM185PE
T-47D
HCC1569
KPL-1
ZR-75-1
CAL-51
DU4475
HCC2157
EFM-19
UACC-812
MDA-MB-435
MCF-7
BT-483
HCC1428
Normal cell line
Luminal cell line
HER2+ cell line
Triple negative cell line
